# Two-dimensional *in situ* metrology of X-ray mirrors using the speckle scanning technique

**DOI:** 10.1107/S1600577515006657

**Published:** 2015-06-06

**Authors:** Hongchang Wang, Yogesh Kashyap, David Laundy, Kawal Sawhney

**Affiliations:** a Diamond Light Source Ltd, Harwell Science and Innovation Campus, Didcot OX11 0DE, UK

**Keywords:** speckle, X-ray optics, metrology

## Abstract

*In situ* metrology overcomes many of the limitations of existing metrology techniques and is capable of exceeding the performance of present-day optics. A novel technique for precisely characterizing an X-ray bimorph mirror and deducing its two-dimensional (2D) slope error map is presented. This technique has also been used to perform fast optimization of a bimorph mirror using the derived 2D piezo response functions. The measured focused beam size was significantly reduced after the optimization, and the slope error map was then verified by using geometrical optics to simulate the focused beam profile. This proposed technique is expected to be valuable for *in situ* metrology of X-ray mirrors at synchrotron radiation facilities and in astronomical telescopes.

## Abstract 2

The two-dimensional slope error of an X-ray mirror has been retrieved by employing the speckle scanning technique, which will be valuable at synchrotron radiation facilities and in astronomical telescopes.

## 1. Introduction   

Nowadays, much advanced scientific research, including X-ray spectroscopy, diffraction and imaging, takes advantage of focused, highly coherent and brilliant X-ray beams generated by modern third-generation synchrotron radiation sources. The successful exploitation of such beams depends to a significant extent on developments in X-ray optics. X-ray mirrors (as an achromatic optic) are widely used at synchrotron radiation facilities for micro- and nano-focusing and the push toward higher spatial resolution requires diffraction-limited and coherence-preserved beams which demand more accurate metrology on X-ray mirrors (Sawhney *et al.*, 2013 ▸). Traditional visible-light metrology techniques such as Fizeau interferometry and long-trace profilometry (LTP) or nanometer optical metrology (NOM) are routinely used for *ex situ* measurement of mirror surface profiles (Alcock *et al.*, 2010 ▸). However, both LTP and NOM can only measure mirror slope errors along one direction at a time. Although Fizeau interferometry can measure two-dimensional (2D) mirror surfaces, it requires a dedicated reference surface for each mirror shape and the measurement accuracy is strongly dependent on the calibration of this reference surface. In addition, the ultimate performance of X-ray mirrors is also affected by the heat load, mechanical clamping or upstream wavefront distortion when they are installed in a beamline environment (Wang *et al.*, 2013 ▸; Rutishauser *et al.*, 2013 ▸). Hence, *in situ* metrology techniques are highly desirable to overcome these limitations and to allow improvements in the performance of X-ray mirrors. In the last two decades, many *in situ* metrology methods have been developed. The pencil-beam technique is simple and easy to set up, but it suffers from low sensitivity and can provide only one-dimensional information (Sutter *et al.*, 2012 ▸; Hignette *et al.*, 1997 ▸). Hartmann sensors can be used to measure 2D wavefronts, but the resolution is limited by the pitch of the micro-lens arrays (Idir *et al.*, 2010 ▸). Recently, grating interferometers have been used for metrology. They give high angular sensitivity but require the use of precision gratings and a complex experimental setup (Wang *et al.*, 2011 ▸, 2014 ▸).

Here, we present a novel method for *in situ* 2D metrology of X-ray mirrors by using the speckle scanning technique. In this technique we mount a sheet of abrasive paper upstream of the mirror under test, and the 2D mirror slope error is measured by scanning the abrasive paper transversely to the X-ray beam. This technique offers both simplicity in terms of experimental implementation and high accuracy for angular sensitivity. We have successfully employed this technique for the optimization of a bimorph mirror, allowing the piezo voltages to be optimized to achieve the smallest focal size.

## 2. Principle   

The technique presented here is based on X-ray near-field speckle, which has also been used in X-ray phase-contrast imaging, coherence measurements and one-dimensional mirror metrology (Berujon *et al.*, 2012*a* ▸,*b* ▸, 2013 ▸, 2014 ▸; Alaimo *et al.*, 2009 ▸; Wang *et al.*, 2015 ▸). When a monochromatic partially coherent beam is passed through a random medium, a 2D speckle pattern is produced by the mutual interference of many waves. Each speckle pattern is unique, and this remarkable property allows its use as wavefront markers. When translating the abrasive paper transversely to the beam axis, the speckle pattern moves across the detector so that the detector records the same speckle at a different pixel position in different frames. Although the one-dimensional (1D) slope error can be retrieved by tracking the speckle displacements (Berujon *et al.*, 2014 ▸), the local defects will be smeared out along the transverse direction of the mirror. In contrast, we have further developed the speckle tracking technique in the present work and obtained a complete 2D slope error map of the mirror surface.

Let the ray position on the mirror surface be 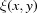. As described earlier, it can be recovered by tracking the speckle displacement. Here, we define the complex field 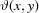 as
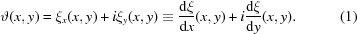
Although 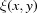 can be calculated by simply performing 1D integration, artefacts will be produced along the direction of the integration due to propagation of statistical errors (Kottler *et al.*, 2007 ▸). In order to perform 2D integration, the two first derivatives of 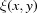, *i.e.* 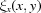 and 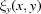, are retrieved using the normalized cross-correlation operation between two neighbouring detector pixels and defined as follows,
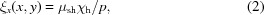


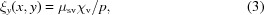
where 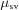 (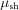) is the abrasive paper scanning step in the vertical (horizontal) direction, 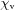 (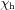) is the vertical (horizontal) displacement of the speckle pattern obtained using the pixel-wise analysis and *p* is the detector pixel size. The speckle pattern is inherently 2D and, if the incidence wavefront is spherical, the speckle is expected to move in both horizontal and vertical directions, even if the abrasive paper is translated only vertically. Therefore, instead of performing two orthogonal scans, the horizontal displacement 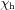 can be retrieved from the same dataset for the vertical scan. In this case, the abrasive paper step size is equal to the pixel size (*p*) of the detector. Hence, equation (2) can be simplified to
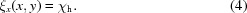
Thereafter, the ray position on the mirror surface 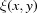 is obtained by using the Fourier transform relation between the derivative and the function as follows,
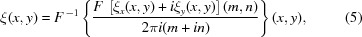
where 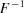 (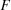) is the inverse (forward) Fourier operations and 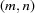 are the variables in the Fourier space corresponding to the real space variables 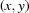 (Kottler *et al.*, 2007 ▸). Once a 2D map of 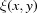 is calculated, the mirror slope map can be recovered iteratively by using the method described by Berujon *et al.* (2014 ▸).

## 3. Experiment and results   

**Figure 1:**
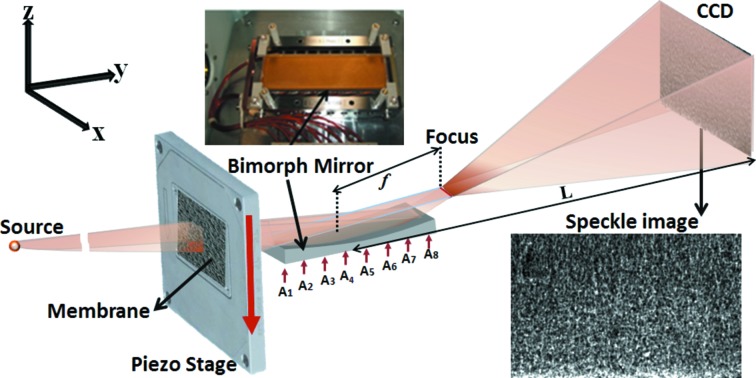
Optical layout of the *in situ* characterization and optimization of a bimorph mirror with eight piezo actuators A1, A2,…, A8.

The experiments were conducted at the test beamline B16 at the Diamond Light Source which takes X-rays from a bending magnet (Sawhney *et al.*, 2010*a* ▸). An X-ray energy of 9.2 keV was selected by a silicon double-crystal monochromator (DCM). As shown in Fig. 1 ▸, the bimorph mirror under test was mounted on a motorized tower, which was located in the experimental hutch at 47 m from the source. The bimorph mirror was made from a silica substrate with a preformed elliptical surface and had eight piezo electrodes cemented underneath to allow the curvature to be varied in order to correct the figure error (Sawhney *et al.*, 2010*b* ▸). Instead of using a phase membrane as in previous work (Berujon *et al.*, 2012*a* ▸,*b* ▸, 2013 ▸, 2014 ▸; Wang *et al.*, 2015 ▸), a sheet of abrasive paper was chosen to increase the speckle contrast. The abrasive paper (FEPA Grit P3000 with average particle diameter of 6.5 µm) was mounted on a piezo stage which was placed 200 mm upstream of the mirror. The speckle pattern was recorded by a 2D detector with an effective pixel size of *p* = 6.4 µm. In order to resolve the speckle features and obtain good angular sensitivity, the detector was located at a large distance *L* = 3.636 m downstream from the mirror. In addition, a high-resolution PCO 4000 CCD camera was mounted on a motorized stage to record the X-ray beam profile at the focus before and after the optimization.

**Figure 2:**
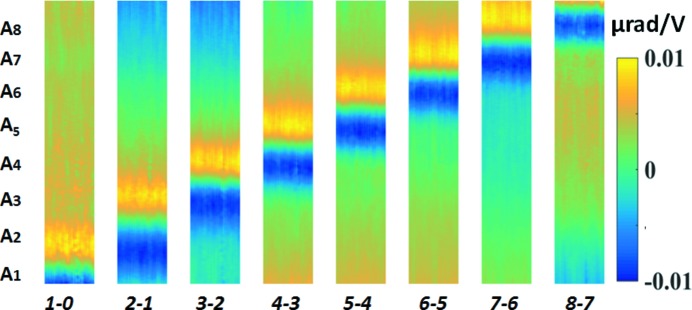
2D plot of the piezo response function of the bimorph mirror. The slope change is induced by applying a fixed voltage to each piezo actuator from first to eighth in sequence. Here 1–0 means the slope response of the first electrode calculated by subtracting the zeroth scan (no voltages applied) from the first scan (voltage applied only to first electrode).

The surface of the bimorph mirror was pre-polished to an ellipse with focal length of *f*_0_ = 0.4 m for X-rays with grazing angle of incidence at the centre of the mirror of θ = 3 mrad. To determine the set of optimum voltages that minimized the slope errors on the mirror, the piezo response function was first measured by collecting a series of images. The first stack of images with a step size of μ = 0.2 µm was acquired with the piezo voltages all set to 0 V. A set of 60 images was collected and the detector acquisition time was 1 s for each image. For the subsequent image stack, the voltage on each electrode was incremented by ν = 400 V, until by the ninth image stack all piezo voltages were at 400 V. Following the procedure described above, the 2D slope map was retrieved from each image stack. Accordingly, the 2D piezo response function of the electrode was calculated by subtracting the values of the slope extracted from the (*j* − 1)th and *j*th image stack. As shown in Fig. 2 ▸, the slope change can be clearly observed at each piezo actuator position. The study of the 2D piezo response function can provide invaluable information about the response of optics to the piezo actuators and will be very valuable for the complex optimization for the X-ray mirror with 2D piezo array, especially used in the astronomical telescope (Atkins *et al.*, 2009 ▸). For the bimorph mirror under study, the piezo actuators could only correct the slope errors along the mirror tangential direction. Therefore, the mirror slope 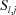 was averaged along the mirror sagittal direction and stored in a 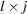 matrix. Here, *l* is the total number of pixels along the mirror tangential direction. Hence, a 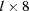 interaction matrix *M*, which defines the response of each electrode per unit voltage change, is constructed from 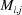 = 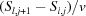, where 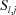 is the slope of the mirror in the *l*th pixel for the *j*th scan.

**Figure 3:**
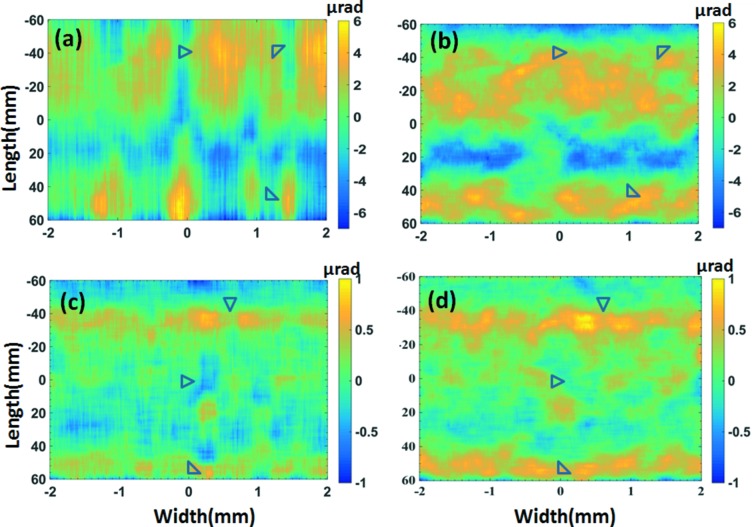
Slope error (*a*) using 1D tracking of speckle and (*b*) after using the proposed method technique at zero voltage (*c*) using 1D tracking of speckle and (*d*) after using the proposed method technique at optimized voltage. The slope error map using the 1D speckle tracking method shows systematic vertical stripes. Triangles mark the region where the artefacts due to 1D integration have been corrected by the proposed method.

The slope error 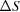 without correction over the whole surface was calculated by fitting an ellipse. The same procedure described by Wang *et al.* (2014 ▸) was employed here, and the required voltage *V*_*n*_ was calculated by multiplying the Moore–Penrose pseudo-inverse matrix *M* ^+^ with the slope error 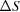 (Signorato *et al.*, 1998 ▸). For comparison, the slope error maps from both 1D integration (*a* and *c*) and 2D integration (*b* and *d*) before and after applying correction voltages are displayed in Fig. 3 ▸. As marked by triangles in Figs. 3(*a*) and 3(*c*) ▸, that 1D integration method shows vertical stripes parallel to the direction of integration which causes systematic errors in the surface error profile of the mirror. On the contrary, the proposed method is superior as it preserves the 2D character of the slope error map as can be seen from Figs. 3(*b*) and 3 ▸(*d*) ▸; the systematic errors are removed and the slope error profile is reproduced in the expected way. These 2D slope error maps also reveal the slope error variation along the sagittal direction (mirror width). This information cannot be accurately detected by the previous 1D technique (Berujon *et al.*, 2014 ▸). The initial slope error with all the piezo voltages set to 0 V was 1.7 µrad (r.m.s.). When the optimized set of piezo voltages were applied, the slope error was dramatically reduced down to 0.4 µrad (r.m.s.). It should be mentioned that 20 pixels in each row were used for calculating the slope error, to improve the signal-to-noise ratio, which gave an equivalent sagittal spatial resolution of 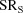 = 0.12 mm. The tangential spatial resolution (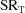) is defined by the detector pixel size (*p*), geometrical magnification ratio 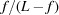 and the mirror grazing incidence angle θ,
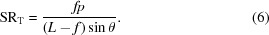
Here *f* is the focal length. In this case, the tangential spatial resolution is about 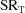 = 0.27 mm. Hence, both the sagittal and tangential spatial resolutions for the measured slope error are in the mid-frequency region. Such information is expected to be extremely valuable for studying the contribution of the small- and large-angle scattering of X-rays from the mirror surface (Harvey *et al.*, 1995 ▸). It may be mentioned that it is very challenging for current *ex situ* metrology techniques to measure the slope error in the mid-frequency region in both the tangential and sagittal directions. The angular sensitivity derived for the 1D slope error measurement can also be applied to the 2D case. Apart from the two predominant parameters *L* and μ, the tracking accuracy η also determines the angular sensitivity of the speckle scanning technique (Berujon *et al.*, 2012*b* ▸; Wang *et al.*, 2015 ▸). The tracking accuracy η is about 0.05 pixel in this study, and it can be further improved by acquiring a few additional images at each abrasive paper position which will increase the signal-to-noise ratio. With μ = 0.25 µm and *L* = 3.636 m, the angular sensitivity for the wavefront error is about 3 nrad. The ultimate accuracy for the absolute slope error is limited by the imperfection of the incoming wavefront.

**Figure 4:**
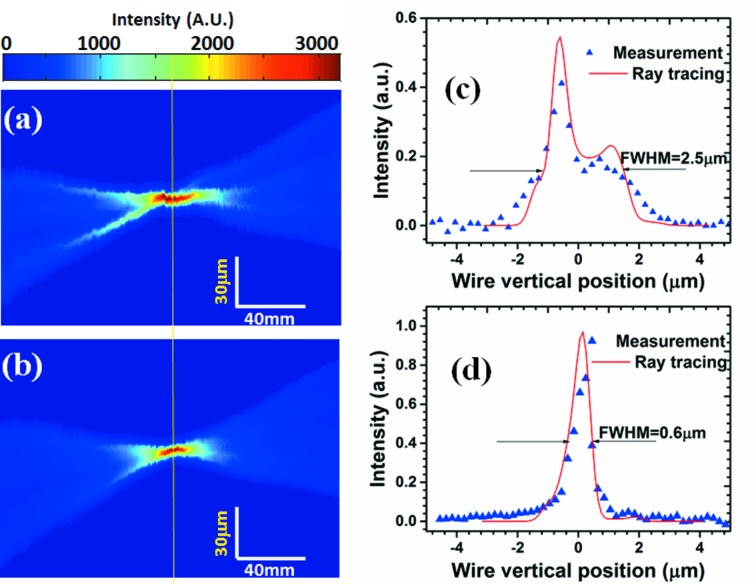
(*a*), (*b*) Intensity profiles as a function of detector distance from the bimorph mirror before and after optimization. (*c*), (*d*) Ray-tracing beam size and the first derivative of the transmission signal from a gold wire scan in the focal plane [yellow line in (*c*) and (*d*)] for the two cases.

To verify the focus optimization, a series of images were taken using a high-resolution PCO 4000 camera which was scanned along the beam direction (*z*) with a 1 mm step size. Figs. 4(*a*) and 4(*b*) ▸ show the intensity profiles around the focal plane of the bimorph mirror. It can be seen that there are strong oscillations on the edge of the beam profile in Fig. 4 ▸(*a*). This is mainly caused by the larger slope error on the mirror before optimization. The beam intensity profile becomes more uniform with a smaller beam size at the focus after optimization as shown in Fig. 4(*b*) ▸. To validate the optimization results, the beam size was also measured by performing a knife-edge scan using a 200 µm-diameter gold wire with the intensity measured by an X-ray diode detector. The measured beam profiles along the lines shown in Figs. 4(*a*) and 4(*b*) ▸ which pass through the focus centre are shown in Figs. 4(*c*) and 4(*d*) ▸, respectively. It shows that the beam size was reduced from 2.5 µm to 0.6 µm after optimization.

In addition, the focused beam profile was calculated from the measured mirror slope error using geometrical ray tracing as follows. If the slope error at position *y* along the mirror surface is 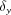 then rays from this point on the mirror will have a distribution at the focal plane centred on position 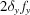, where 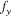 is the distance to the focus. The profile will be given by the demagnified profile of the source. If the incident intensity is assumed to be uniformly distributed along the mirror, the focus profile can be determined by summing the contribution to the profile from each point along the mirror. The calculation is shown as the solid lines in the figures and there is good agreement with the beam profile measured by the edge scan which is shown as points in the figure.

## 4. Summary   

In summary, we have demonstrated that the speckle scanning technique can be used for the measurement of 2D slope error profiles of X-ray mirrors. It can provide valuable information on optical aberrations of the X-ray aspherical mirrors, such as ellipsoidal and toroid mirrors, which are difficult to obtain using conventional *ex situ* visible-light metrology techniques. In contrast to other *in situ* techniques such as the pencil-beam technique or Hartmann sensors, the X-ray speckle scanning technique is fast, compact and accurate. The advantage of our technique is that it can generate 2D slope error maps by performing only 1D scans. The successful demonstration of mirror optimization shows that this metrology technique can be potentially used for characterization and optimization of advanced X-ray mirrors in astronomical telescopes.
